# Analysis of risk factors for painful diabetic peripheral neuropathy and construction of a prediction model based on Lasso regression

**DOI:** 10.3389/fendo.2024.1477570

**Published:** 2024-10-22

**Authors:** Zikai Yu, Sue Zhao, Jing Cao, Hebin Xie

**Affiliations:** The Affiliated Changsha Central Hospital, Hengyang Medical School, University of South China, Changsha, China

**Keywords:** painful diabetic peripheral neuropathy, type 2 diabetes mellitus, diabetic, risk, LASSO regression

## Abstract

**Objective:**

To evaluate the prevalence and risk factors of painful diabetic peripheral neuropathy (PDPN) in patients with type 2 diabetic peripheral neuropathy (DPN) in Hunan Province, and establish and verify the prediction model.

**Methods:**

This was a retrospective study involving 4908 patients, all patients were randomly divided into the training dataset(3436 cases)and the validation dataset (1472 cases) in a ratio of 7:3. Electroneurogram, clinical signs,and symptoms were used to evaluate neuropathy. Least absolute shrinkage and selection operator (LASSO) regression was used to select the optimal factors, and multifactorial logistic regression analysis was used to build a clinical prediction model. Calibration plots, decision curve analysis (DCA), and subject work characteristic curves (ROC) were used to assess the predictive effects.

**Result:**

The prevalence of PDPN was 33.2%, and the multivariate logistic regression model showed that peripheral artery disease, duration of diabetes, smoking, and HBA1c were independent risk factors for PDPN in patients with type 2 diabetes. ROC analysis results showed that the AUC of the established prediction model was 0.872 in the training dataset and 0.843 in the validation dataset. The calibration curve and decision curve show that the model has good consistency and significant net benefit.

**Conclusion:**

33.2% of DPN patients had PDPN in Hunan Province, China. Peripheral artery disease, duration of diabetes, smoking, and HBA1c are risk factors for PDPN in patients with type 2 diabetes. The prediction model is based on the above factors, which can well predict the probability of PDPN.

## Introduction

Diabetes mellitus (DM) is a serious threat to human health and a global healthcare problem with high economic costs. According to a recent report by the International Diabetes Federation, by 2021, the number of adults with diabetes worldwide had reached 500 million, accounting for 11.0% of the global population that year (1). The 2019 Global Burden of Disease (GBD) shows that deaths due to diabetes increased by 147.1% in 2019 compared to 1990 (2). China has the highest prevalence of diabetes in the world, with 90% of the cases being type 2 DM(T2DM) (3).

Diabetes peripheral neuropathy (DPN) is the most common complication of T2DM, with a prevalence of 30.0% to 90.0% (4). Around 21.0%-53.7% of people with DPN have painful DPN(PDPN), but many are unaware of the existence of this complication (5, 6). A national study in Germany found that about 61.0% of patients with painful peripheral neuropathy had not been diagnosed before the study (7). A similar situation exists in primary hospitals in China. PDPN is a typical neuropathic pain, patients often have symmetrical spontaneous pain in distal limbs, and allergy to pain. In severe cases, persistent pain is associated with nighttime aggravation, and more than half of patients suffer from sleep disorders and depression, leading to a serious impact on the quality of life of the patients and reducing work efficiency (8). A clinical study reported that compared with diabetic patients without DPN or painless DPN, patients with PDPN had a higher disease burden (9).

The pathogenesis of PDPN is still unclear, but it is widely believed to be closely related to glucose metabolism disorder, inflammatory response, oxidative stress, polyol bypass activation, mitochondrial damage, and glial cell activation (10, 11). A studies have shown that cell suppressive drugs can release histamine (causing allergic reactions), which can cause swelling and degeneration of axons, and increase the risk of peripheral neuropathy (12, 13). In addition, the results of a clinical study found that the combination of fenforamine and alpha-lipoic acid was protective against the progression of diabetic neuropathy (14). Callaghan et al. found that glycemic control may prevent or delay the development of diabetic peripheral neuropathy (15). Nevertheless, other studies have shown that glycemic control was not enough to prevent and improve PDPN, especially in people with type 2 diabetes (5). Jensen et al. reported that about 40% of PDPN patients were not effectively treated, and those who did receive treatment had unsatisfactory results (16). Therefore, the early detection and prevention of PDPN is particularly important. However, there are fewer studies on the prevalence and risk factors of PDPN in China, and there are no studies in Hunan Province. Hence, the objective of this study was to assess the prevalence of PDPN and associated risk factors in patients with type 2 diabetic peripheral neuropathy in Hunan Province, China, and to establish and evaluate predictive models.

## Methods and patients

### Research design and patients

This study was a retrospective and based on the 4908 patients with T2DM-induced DPN diagnosed from January 2020 to December 2023 in the Changsha Central Hospital. The eligible subjects were aged from 18 to 70 years and had been with diagnosed T2DM-induced DPN. History of psychiatric, cancer, cervical or lumbar spine lesions(nerve compression, spinal stenosis), cerebral infarction, lower extremity vascular occlusion, alcoholism, connective tissue disease, and those who were pregnancy or lactation were excluded.

The study was approved by the Ethics Review Committee of the Changsha Central Hospital, and the study adhered to the principles of the Declaration of Helsinki.

### Data gathering

General demographic data, clinical data, and laboratory results were the main types of data collected for this study, including age, gender, marital status, payment method, occupation, smoking, height, weight and body mass index (BMI), clinical pathway, duration of diabetes, hospital admissoin days, systolic blood pressure (SBP), diastolic blood pressure (DBP), complications of diabetes, other comorbidities, fasting blood glucose (FBG), glycated hemoglobin (HbA1c), d-dimer (DD), albumin (ALB), international normalized ratio (INR), activated partial thromboplastin time (APTT), thrombin time (TT), prothrombin time (PT), fibrinofen (Fib), serum creatinine (Scr), low-density lipoprotein (LDL), triglycerides (TG), high-density lipoprotein (HDL), and total cholesterol (TC) were obtained from the electronic medical record system.

### Assessment of DPN and PDPN

Diagnostic criteria for DPN:① Clear history of diabetes; ② Neuropathy at the time of or after the diagnosis of diabetes mellitus; ③ Clinical symptoms of neuropathy, such as pain, numbness, sensory abnormalities, and abnormalities in any one of the 5 tests (ankle reflexes, vibration sensation, pressure sensation, temperature sensation, and pinprick pain sensation); if there are no clinical symptoms, abnormalities in any two of the 5 tests can be diagnosed (17).; ④ Electroneurogram was performed in all patients; ⑤ Exclude other causes of peripheral nerve injury, such as neurogenic cervical and lumbar spondylosis, Guillain-Barre Syndrome, cerebral infarction, and drugs (18, 19). PDPN should meet the above diagnostic criteria of DPN, and the pain is bilateral, below the knee, often worse at night, and not related to exertion (20).

### Electroneurogram

Electroneurogram examine nerve conduction function (model:MEDCOM; Zhuhai Maikang Technology Co., LTD.). Place the patient in a supine position, and the skin temperature of the patient was kept at 32 ~ 35°C during the measurement. The median nerve (MN) sensory and motor conduction velocity, common peroneal nerve (CPN) sensory and motor conduction velocity, and MN-F and CPN-H reflex latency were respectively detected. The final recorded value was the average value of three consecutive tests.

### Statistical analysis

R, a software environment (R version 3.3.2) and STATA software version 13.1 (Stata Corp., College Station, TX) were used for statistical computing. Normal distribution data were presented as mean ± standard deviation,and compared using student’s t-test; nonnormally distributed variable were presented by the median(p25,p75), and compared by Mann-Whitney U test; categorical data were presented as number (rate), and Pearson χ^2^ test was used to compare. Set the number of seeds (123) and randomly divide the sample into modeling and validation groups in a ratio of 7:3.Variables with statistical significance in the univariate analysis were dimensional reduction using least absolute shrinkage and selection operator (LASSO)regression, and using cross-validation to select λ ;Variables with non-zero regression coefficients at lambda.1-s were included in the Logistic regression analysis, constructed LASSO-Logistic homoscedastic prediction model, and plotted nomogram. The receiver operating characteristic (ROC) curve was evaluated accuracy of the model. The model was verified by the Bootstrap resampling method (B=1 000). Calibration was assessed using calibration curves and Hosmer-Lemeshow goodness-of-fit tests, and clinical utility was assessed using decision curve analysis (DCA). A two-tailed *P* value of <0.05 was regarded as statistically significant.

## Results

### Prevalence of PDPN and frame design

A total of 4908 patients with DPN were included in this study, with 1627 patients having PDPN, showing a prevalence of 33.2% in the entire study population.We randomly divided 4908 patients into a training dataset (3436 cases) and a validation dataset (1472 cases) in a 7:3 ratio. Within the training dataset, patients were further categorized into PDPN group (1127 cases) and non-PDPN group (2309 cases).

### Characteristics of PDPN and non-PDPN patients in the training dataset

We found statistically significant differences in marital status, mode of payment, occupation, BMI, and smoking between the two groups based on demographic information (*p*<0.05). Comparison of clinical data, including clinical pathway, duration of diabetes, peripheral vascular disease of type 2 diabetes, combination of two or more diabetic complications, hypertension, and surgical history differences were statistically significant (*P*<0.05). Moreover, by analyzing the results of laboratory tests, we found that there were differences between the two groups of FBG, HbA1c, DD, Scr, LDL, and TC (*p*<0.05). As shown in **Tables 1**, **2**.

**Table 1:** Comparison of characteristics between DPNP and non-DPNP patients in the training dataset (categorical variable).

Variables	Category	No Painful diabetic neuropathy (n=2309)	Painful diabetic neuropath (n=1127)	*χ^2^*	*p*
Gender	Male	1260 (54.57)	634 (56.26)	0.871	0.351
	Female	1049 (45.43)	493 (43.74)		
Marital status	Unmarried	46 (1.99)	24 (2.13)	8.054	0.045
	Married	2139 (92.64)	1066 (94.59)		
	Divorced	99 (4.29)	27 (2.4)		
	Widowed	25 (1.08)	10 (0.89)		
Payment method	Medical insurance for urban employees	1687 (73.06)	771 (68.41)	14.928	0.011
	Medical insurance for urban residents	308 (13.34)	172 (15.26)		
	New rural cooperative medical	110 (4.76)	76 (6.74)		
	Self-payed	135 (5.85)	84 (7.45)		
	At state expense	39 (1.69)	14 (1.24)		
	Commercial insurance	30 (1.3)	10 (0.89)		
Occupation	Professional and technical personnel	181 (7.84)	91 (8.07)	15.859	0.015
	Civil servant	46 (1.99)	17 (1.51)		
	Worker	63 (2.73)	22 (1.95)		
	Farmer	159 (6.89)	51 (4.53)		
	Retirees	1371 (59.38)	661 (58.65)		
	Self-employed individual	38 (1.65)	19 (1.69)		
	Others	451 (19.53)	266 (23.6)		
BMI	<24kg/m^2^	2104 (91.12)	996 (88.38)	6.470	0.011
	≥24kg/m^2^	205 (8.88)	131 (11.62)		
Smoking	No	1798 (77.87)	708 (62.82)	86.870	<0.001
	Yes	511 (22.13)	419 (37.18)		
Clinical pathway	Completed	2082 (90.17)	981 (87.05)	7.636	0.006
	Drop out	227 (9.83)	146 (12.95)		
Diabetic nephropathy in patient with T2DM	No	1597 (69.16)	794 (70.45)	0.594	0.441
	Yes	712 (30.84)	333 (29.55)		
Diabetic retinopathy in patient with T2DM	No	1742 (75.44)	883 (78.35)	3.546	0.060
	Yes	567 (24.56)	244 (21.65)		
Peripheral arterial disease in patients with T2DM	No	2196 (95.11)	906 (80.39)	186.89	<0.001
	Yes	113 (4.89)	221 (19.61)		
Combination of two or more complications of diabetes	No	1249 (54.09)	545 (48.36)	9.980	0.002
	Yes	1060 (45.91)	582 (51.64)		
Hypertension	No	891 (38.59)	390 (34.61)	5.139	0.023
	Yes	1418 (61.41)	737 (65.39)		
History of surgery	No	2172 (94.07)	1033 (91.66)	6.999	0.008
	Yes	137 (5.93)	94 (8.34)		

**Table 2:** Comparison of characteristics between DPNP and non-DPNP patients in the training dataset (continuous variable).

Variables	No Painful diabetic neuropathy (n=2309)	Painful diabetic neuropath (n=1127)	*t/z*	*p*
Age, year (mean ± SD)	63.54 ± 11.98	64.18 ± 11.18	-1.503	0.133
SBP, mmHg (mean ± SD)	123.41 ± 17.50	123.73 ± 17.31	-0.504	0.614
DBP, mmHg (mean ± SD)	76.83 ± 12.37	76.08 ± 10.90	1.737	0.082
Duration of diabetes, year,Median (IQR)	7.60 (2.90,12.40)	13.90 (8.90,22.90)	-22.77	<0.001
Hospital admissoin days, day [Median (IQR)]	9.00 (7.00,12.00)	9.00 (7.00,12.00)	-8.409	0.244
FBG, mmol/L (mean ± SD)	7.45 (6.10,7.45)	7.95 (6.05,9.90)	-2.286	<0.001
HbA1c, % (Median (IQR))	7.38 (6.40,7.90)	8.54 (6.45,10.51)	-12.700	<0.001
DD,mg/L (mean ± SD)	0.52 ± 0.19	0.54 ± 0.23	-2.194	0.028
ALB,g/L (mean ± SD)	35.57 ± 11.41	34.84 ± 11.47	1.741	0.082
INR, (mean ± SD)	1.09 ± 0.26	1.08 ± 0.23	1.362	0.173
APTT,s (mean ± SD)	27.70 ± 9.07	27.40 ± 6.25	1.018	0.309
TT,sec (mean ± SD)	16.59 ± 5.97	16.59 ± 4.54	-0.015	0.988
PT,sec (mean ± SD)	7.73 ± 4.67	7.52 ± 4.73	1.260	0.208
Fib,g/L (mean ± SD)	2.98 ± 0.99	2.97 ± 1.04	0.129	0.897
Scr,μmol/L,Median (IQR)	64.01 (49.67,77.54)	65.50 (51.44,80.33)	-2.993	0.003
LDL, mmol/L,Median (IQR)	2.51 (2.01,3.10)	2.61 (1.99,3.47)	-3.478	0.001
TG, mmol/L,Median (IQR)	1.71 (1.24,2.21)	1.75 (1.24,2.38)	-1.134	0.257
HDL, mmol/L,Median (IQR)	1.21 (1.12,1.49)	1.25 (0.98,1.69)	-1.623	0.105
TC, mmol/L,Median (IQR)	4.44 (4.12,5.34)	4.60 (4.00,5.60)	-2.259	0.024

Normal distribution data were presented as mean ± standard deviation,and compared using student’s t-test; nonnormally distributed variable were presented by the median (IQR),and compared by Mann-Whitney U test.

### Variable selection and modeling of PDPN influencing factors

**Figure 1:**
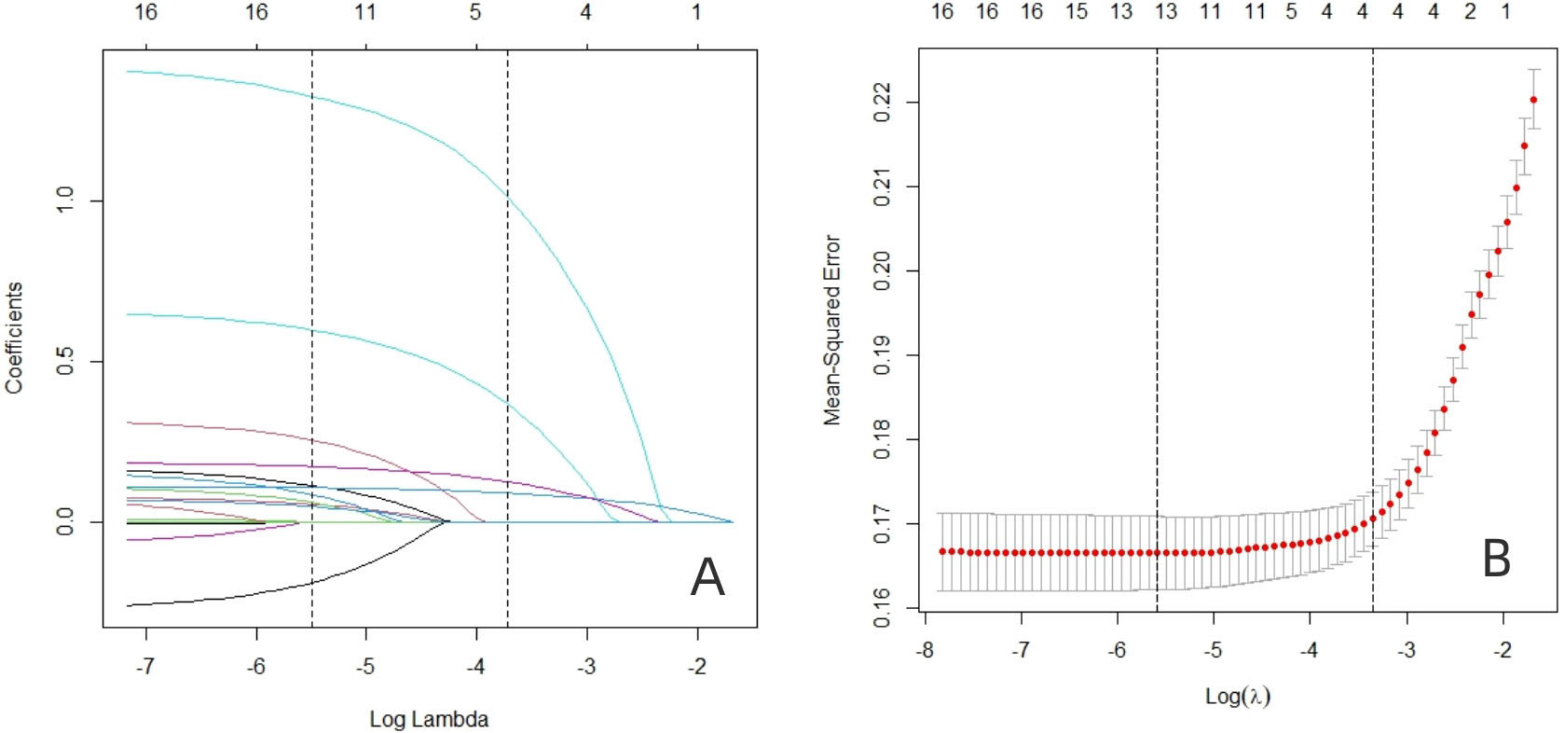
The predictors were selected using LASSO regression. **(A)** Variation characteristics of variable coefficient. The abscissa represents the value of the parameter log(λ), and the ordinate represents the coefficient of the independent variable. Finally, the coefficients of all independent variables are compressed to 0, and the later the independent variable becomes 0, the greater the contribution to the model. The dashed line on the left represents the value of the parameter log(λ) where the model error is smallest, and the dashed line on the right represents the value of the parameter log(λ) when the model error is amplified by one standard error. **(B)** 10-fold cross-validation of adjusted parameters. The abscissa represents the value of the parameter log(λ), and the ordinate represents the mean square error of the model.

**Figure 2:**
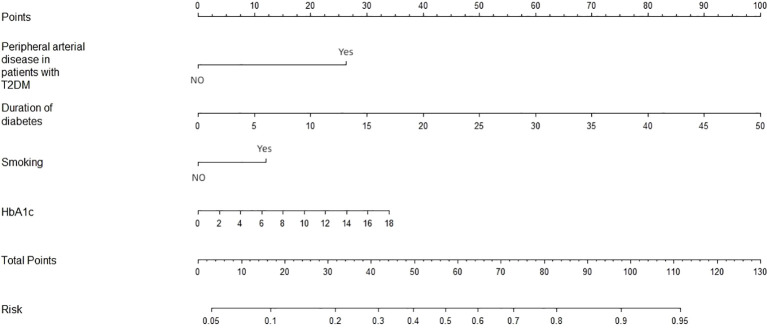
Nomogram was plotted based on four optimal predictorsto predict PDPN in the training dataset.

In the training dataset, the 17 variables with statistically significant differences in **Table 1** (marital status, payment method, occupation, BMI, somking, clinical pathway, duration of diabetes, peripheral vascular disease of type 2 diabetes, combination of two or more diabetic complications, hypertension, surgical history, FBG, HbA1c, DD, Scr, LDL, and TC)were used as independent variables. Lasso analysis was performed using glmnet in R software (**Figures 1A, B**), and the results showed that the model performance was optimal when the penalty coefficient λ = 0.004, which finally screened out duration of diabetes, peripheral arterial disease in patients with type 2 diabetes, smoking, and HbA1c were finally selected as four potential predictors. Subsequently, further logistic regression analysis showed that duration of diabetes, peripheral arterial disease in patients with type 2 diabetes, smoking, and HbA1c were associated with PDPN (**Table 3**). In addition, we plotted the nomogram based on the results of multifactor logistic regression for a more intuitive estimation of individual risk (**Figure 2**).

**Table 3:** Multivariate logistic regression analysis of factors associated with PDPN among T2DM patients.

Variables	OR	95% CI	*p*
Peripheral arterial disease in patients with T2DM	4.222	3.21-5.55	<0.001
Duration of diabetes	1.126	1.11-1.14	<0.001
Smoking	1.953	1.63-2.35	<0.001
HbA1c	1.202	1.16-1.24	<0.001

### Validation of a line graph model of factors influencing the prevalence of PDPN

**Figure 3:**
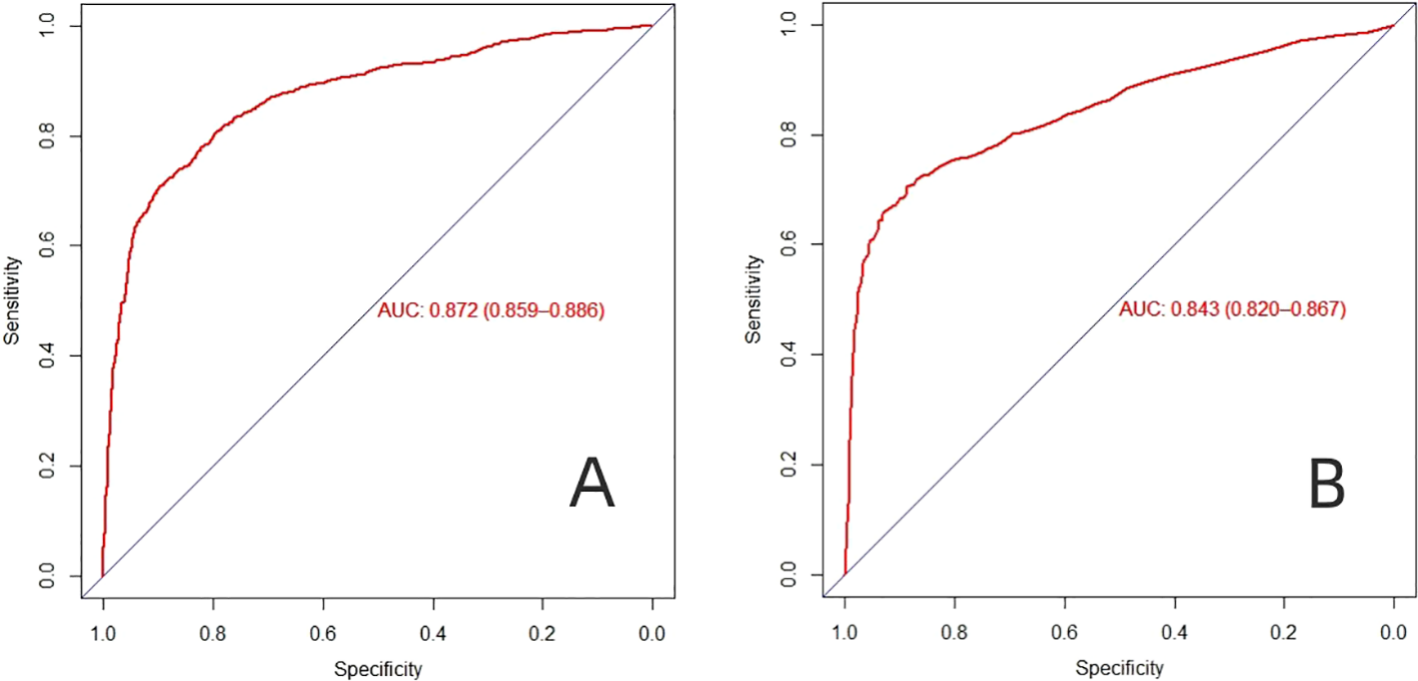
The AUC-ROC curves of predictive nomogram in **(A)** the training dataset and **(B)** the validation dataset.

**Figure 4:**
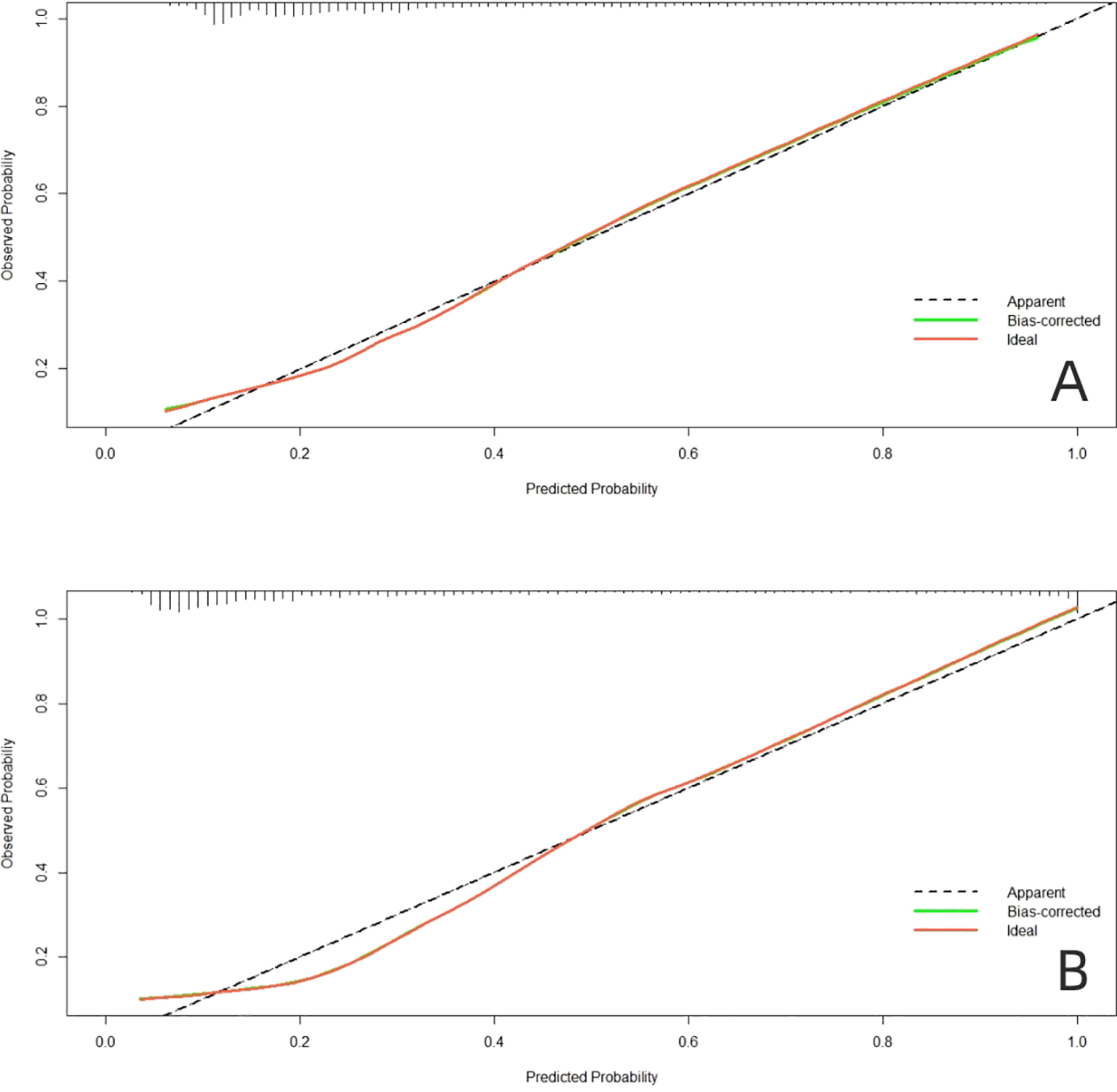
Calibration plots of the nomogram in **(A)** the training dataset and **(B)** the validation dataset.

The AUC of the model in the training dataset (**Figure 3A**) and validation dataset (**Figure 3B**) were 0.872 (95% *CI*: 0.859-0.886), and 0.843 (95% *CI*: 0.820-0.867), indicating that the model has high predictive value. The calibration curve shows that the predicted probability of the model is in good agreement with the actual incidence (**Figure 4A, B**). In addition, the Hosmer Lemeshow test (training dataset *p*=0.671; validation dataset *p*=0.794) showed good probabilistic agreement between the predictive model and the actual probabilities.

### Clinical practicability of nomogram model

**Figure 5:**
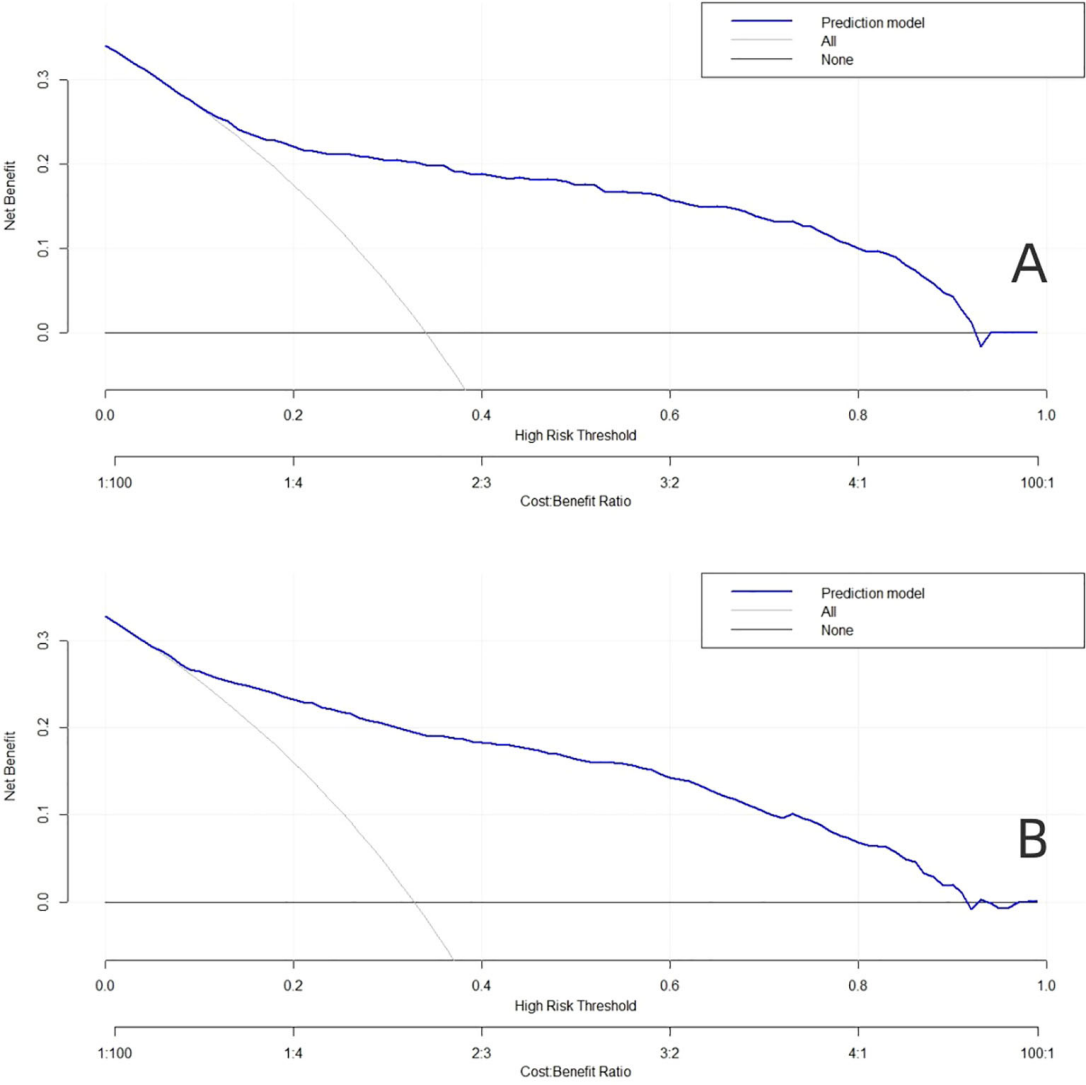
Decision curve analysis (DCA) for the predictive model. **(A)** The training dataset. **(B)** The validation dataset.

When the high risk threshold is 0.1-0.9, the net return rate is >0, and the model has clinical significance. In addition, the smaller the high-risk threshold, the greater the net return, indicating that the model has better clinical benefits (**Figure 5A, B**).

## Discussion

The current study aimed to evaluate the prevalence and risk factors of PDPN among patients with type 2 diabetes. In our study, the prevalence of PDPN in DPN patients with type 2 diabetes mellitus was 33.2%, and the results of this study are similar to those of previous studies. The prevalence was 34.5% in a study conducted at two national diabetes centers in Qatar (21). Furthermore, in recent studies in the Arabian Gulf region, a similar prevalence of 43.3%was reported (22). However, a study conducted in Saudi Arabia was found to be 65.3% (23). The difference in prevalence rates in a diverse country is considered due to the different study subjects, study methods, and diagnostic criteria used to define PDPN.

Diabetic complications are the main cause of death and disability in diabetic patients (24). Pararajasingam et al. reported that microvascular/macrovascular complications occur early in patients with diabetes, and some patients with type 2 diabetes developed microvascular/macrovascular complications early in diagnosis (25). The complications of diabetes and other complications of diabetes are interdependent, rather than independent of each other. Previous studies have shown that the most common microvascular complications of diabetes such as diabetic nephropathy and diabetic retinopathy are risk factors for PDPN (26). Another study reported that vascular complications such as hypertension, peripheral arterial disease, and a history of cerebrovascular accident were associated with PDPN; patients with PDPN have more comorbidities than those with non- PDPN (27, 28). In addition, a study in Taiwan showed that peripheral arteriosclerosis increases a patient’s risk of developing PDPN by 2.5 times (29).

In the study by Çelik et al. showed that smoking affects the course of concomitant diseases (30). The results of a recent study show that diabetics who smoke are at a higher risk of developing neuropathy, and heavy smokers have poorer nerve conduction (31). The results of a cross-sectional study reported that smoking was a risk factor for diabetic painful neuropathy (21), which is consistent with our findings.

Duration of diabetes and HbA1c was associated with PDPN in our study. Baxi et al. reported that the duration of diabetes and glycemic control were significantly correlated with neuropathy, and the prevalence increased with the duration of diabetes (26). Furthermore, a cross-sectional study showed that increased postprandial blood glucose exposure, such as patients with higher HbA1c levels (≥7%) and near-normal average FPG, was associated with the risk of developing diabetic painful neuropathy (32). Although the exact mechanism is unknown, it may be due to the activation of multiple biochemical pathways that induce neuronal oxidative stress in a state of sustained hyperglycemia, leading to impaired neuronal metabolism and DNA damage and DNA damage (33). In addition, previous study showed that fibrinogen (FIB),serum albumin (ALB) was an independent risk factor for the development of PDPN, and have a greater predictive potential for PDPN development in T2DM compared with HbA1c (34). However, this trend was not observed in our study.

At present, the screening variables of many prediction models are mainly based on single factor analysis, and the prediction ability of different populations is not ideal. Lasso regression method introduces penalty coefficient into model estimation, which can effectively deal with overfitting and multicollinearity problems of various study variables, and can obtain higher model generalization ability and accuracy (35, 36). In this study, the dimensionality of predictors was reduced by LASSO regression, then Logistics regression was used to screen the predictors, and nomogram was constructed. Training sets (AUC, 0.872) and validation sets (AUC, 0.843), nomogram has good overall predictive performance and can help physicians identify patients at higher risk of PDPN. At the same time, our graph also has good calibration ability. In the training dataset, the predicted risk of PDPN is close to the actual risk, and it is further verified in the validation dataset. The clinical diagnostic value of this nomogram was evaluated by DCA, the results show that the nomogram model can gain benefits under a wide range of threshold probabilities.

There are some limitations to our study. Firstly, the current study was retrospective, and the causal relationship between potential risk factors and PDPN could not be verified. Secondly, there is no global uniform diagnostic criteria for PDPN, and different diagnostic criteria may have an impact on the assessment of prevalence. In addition, some literature suggested that postprandial blood glucose level might be more closely related to the development of PDPN, and we did not collect data on this part (32). Therefore, the above questions will be further refined in subsequent prospective cohort studies.

In this study, the prevalence of PDPN in DPN patients with type 2 diabetes was 33.2%, and Lasso-Logistic regression analysis showed that peripheral artery disease, diabetes duration, smoking, and HBA1c are risk factors for PDPN in type 2 diabetes. Based on the above factors, the established prediction model has a good ability to predict the risk of PDPN, which is helpful for clinicians to identify high-risk groups and carry out targeted early intervention.

## Data Availability

The datasets presented in this article are not readily available because the data came from the electronic medical record system of our hospital, which was a single-center study. Requests to access the datasets should be directed to Jing Cao 523060736@qq.com.
